# Imatinib Mesylate for the Treatment of Canine Mast Cell Tumors: Assessment of the Response and Adverse Events in Comparison with the Conventional Therapy with Vinblastine and Prednisone

**DOI:** 10.3390/cells11030571

**Published:** 2022-02-07

**Authors:** Thais Rodrigues Macedo, Genilson Fernandes de Queiroz, Thaís Andrade Costa Casagrande, Pâmela Almeida Alexandre, Paulo Eduardo Brandão, Heidge Fukumasu, Samanta Rios Melo, Maria Lucia Zaidan Dagli, Ana Carolina B. C. Fonseca Pinto, Julia Maria Matera

**Affiliations:** 1 Department of Surgery, School of Veterinary Medicine and Animal Science, University of São Paulo, São Paulo 05508-010, Brazil; tharmacedo@gmail.com (T.R.M.); samymelo@usp.br (S.R.M.); anacarol@usp.br (A.C.B.C.F.P.); materajm@usp.br (J.M.M.); 2 Department of Animal Science, Federal Rural University of Semi-Arid, Mossoró 59625-900, Brazil; 3 Master’s and Doctor’s Degree Program in Industrial Biotecnology at Positivo University, Curitiba 81280-330, Brazil; thaiscosta@yahoo.com.br; 4 Department of Preventive Veterinary Medicine and Animal Health, School of Veterinary Medicine and Animal Science, University of São Paulo, São Paulo 05508-010, Brazil; pama.alexandre@gmail.com (P.A.A.); paulo7926@usp.br (P.E.B.); 5 Laboratory of Comparative and Translational Oncology (LOCT), Department of Veterinary Medicine, Faculty of Animal Science and Food Engineering, University of Sao Paulo, Pirassununga 13635-900, Brazil; fukumasu@usp.br; 6 Laboratory of Experimental and Comparative Oncology, Department of Pathology, School of Veterinary Medicine and Animal Science, University of São Paulo, São Paulo 05508-010, Brazil; mlzdagli@usp.br

**Keywords:** c-KIT, imatinib mesylate, immunohistochemistry, mast cell tumor, prednisone, vinblastine

## Abstract

Mast cell tumors (MCTs) are common neoplasms in dogs, and treatments for these diseases include surgery, polychemotherapy and targeted therapy with tyrosine kinase inhibitors. This study aimed to evaluate the response and the adverse events of treatment with imatinib mesylate (IM) compared to conventional therapy using vinblastine and prednisolone (VP) in canine cutaneous MCTs. Twenty-four dogs were included in the study; 13 animals were treated with IM and 11 with VP. Tumor tissue samples were submitted for histological diagnosis, grading and KIT immunostaining. The response to treatment was assessed by tomographic measurements according to VCOG criteria. Adverse events were classified according to VCOG-CTCAE criteria. The IM and VP groups had dogs with similar breeds, gender, ages, MCT localization, WHO stages and lymph node metastasis profiles. Most MCTs were grade 2/low and had KIT- patterns 2 and 3. The objective response rate (ORR) was significantly higher (30.79%) in the IM group then in VP group (9.09%). Adverse events (AE) in IM group were all grade 1, significantly different from VP. In conclusion, IM presented better ORR and less severe adverse events when compared to VP, representing a suitable option for the treatment of low-grade canine MCTs.

## 1. Introduction

Mast cell tumors (MCTs) are hematopoietic neoplasms that commonly occur in dogs, accounting for 7–21% of skin tumors reported in this species [1,2]. Recently, canine and human mast cell neoplasms were compared, and the importance of comparative oncology was highlighted: human systemic mastocytosis and canine mast cell tumors share many characteristics such as diagnostic approaches, c-KIT mutations, and even treatment modalities [3]. Most canine mast cell tumors exhibit different mutations in the c-KIT gene, including internal tandem duplications in the juxtamembrane region [4,5], which result in the constitutive activation of KIT, leading to increased and uncontrolled cell proliferation.

Canine MCTs vary widely in terms of their biological behaviors, ranging from nearly benign tumors to highly invasive and metastatic tumors [6]; most authors affirm that these tumors must always be considered malignant. Two histological grading systems were proposed for canine MCT. In Patnaik’s system, MCTs are categorized as grades 1, 2, or 3, so that grade 3 tumors correspond to more aggressive tumors with greater metastatic potential, and are therefore more concerning from a clinical perspective [7]. The more recent Kiupel two-tiered grading system categorizes MCT into high- or low-grade tumors [8]. Histologic grade, location, and c-KIT mutation status are well-established prognostic factors that differentiate between high- and low-grade MCTs [9,10].

The treatment of dogs with MCT consists of either polychemotherapy or tyrosine kinase inhibitors such as toceranib, masitinib, or, less frequently, imatinib [11,12,13]. The treatment decision is based on the clinical and histopathological diagnoses, and on the stage of the disease. The treatment options for mast cell tumors are: surgery, chemotherapy, radiation therapy, or combined treatment. Surgical excision is the treatment of choice for mast cell tumors, which present as single masses located in areas that allow wide excision, with or without the involvement of regional lymph nodes [14]. Mast cell tumors are very invasive and wide surgical margins are indicated to treat these diseases. In cases of multiple tumors, inoperable tumors or the presence of distant metastases, other treatment modalities are indicated [15]. Chemotherapy for mast cell tumors is indicated for the treatment of high-grade tumors in advanced clinical stages, for debulking or to prevent local recurrence in cases of incomplete excisions. [16]. The standard chemotherapy protocol for the treatment of mast cell tumors is the association of vinblastine with prednisone [17].

Receptors with tyrosine kinase activity (RTKs) are widely investigated cell proteins that are often dysregulated in humans and animals with neoplastic diseases [18,19]. The heterogeneous expression of KIT, in addition to VEGFR-2 and PDGFRB, is reported in canine MCT [20,21].

Imatinib mesylate (IM) is a mesylate salt of imatinib, a tyrosine kinase inhibitor with known antineoplastic activity. Imatinib binds to an intracellular pocket located within tyrosine kinases (TK), thereby inhibiting ATP binding, phosphorylation, and the subsequent activation of growth receptors and their downstream signal transduction pathways. This agent inhibits RTKs encoded by c-KIT and platelet-derived growth factor receptor (PDGFR) oncogenes. Imatinib is a selective RTK inhibitor used to treat gastrointestinal stromal tumors in humans [22] and MCT in dogs [11,12,13], as these tumors show abnormal constitutive tyrosine kinase expressions, thereby leading to dysregulated cell growth [5].

IM is used to treat canine MCTs [11,12,13]; however, the efficacy of this agent relative to conventional chemotherapy with vinblastine and prednisone (VP) has yet to be determined. Therefore, this study aimed to evaluate the response to treatment of imatinib mesylate (IM) in comparison to conventional therapy with vinblastine and prednisolone (VP) in dogs with MCT. This study also aimed to compare the adverse events of IM and VP treatments.

## 2. Material and Methods

### 2.1. Ethical Approval, Canine Patients and Inclusion Criteria

This study was approved by the Committee of Ethics on the Use of Animals (CEUA) of the School of Veterinary Medicine and Animal Science of the University of Sao Paulo, FMVZ—USP (process number 2092/2010). The dog owners signed informed consent forms.

A total of 24 client-owned dogs were recruited at the Small Animal Hospital of the School of Veterinary Medicine and Animal Science of the University of São Paulo (FMVZ-USP). The inclusion and exclusion criteria are listed in Table 1.

### 2.2. Diagnosis and Staging

Clinical evaluation and staging included collection of complete data related to medical history, physical examination, complete blood count (CBC; including differential and platelet counts), serum biochemistry, urinalysis, transabdominal ultrasound, and regional lymph node cytology, if feasible.

The diagnosis was made based on the histological analysis of punch biopsy specimens. Tumor samples were collected for histological and immunohistochemical analyses. These were fixed in 10% formalin and routinely processed for inclusion in paraffin. The 5 um sections were positioned in glass slides and submitted to staining with hematoxylin and eosin for diagnosis. Histological slides were examined by a single veterinary pathologist (M.L.Z.D.).

In dogs with multiple MCTs, the largest tumor was selected for grading. Tumors were graded according to both the Patnaik [7] and Kiupel [8] grading systems.

Tumor samples were subjected to DNA and RNA extractions for analysis of c-kit mutations (PCR amplifications and sequencing) and semi-quantitative expression of c-kit and c-kit ligand by Real-Time PCR. Results were not conclusive and, therefore, not included in this study.

### 2.3. Immunohistochemistry

Tissue sections of canine cutaneous MCTs were used for the immunohistochemical evaluation of KIT protein localization, as previously described [22].

To prepare the slides for immunohistochemistry, 5 μm thick sections were obtained from the paraffin blocks, adhered to silanized slides, dewaxed and rehydrated. The recovery of antigens for KIT was performed by heating the histological sections in a 1% citrate buffer solution, pH 6.0, for 3.5 min in a pressure cooker. After cooling, the slides were treated with a block of endogenous peroxidase for 30 min in a 6% hydrogen peroxide solution, followed by washing the slides in running water for 10 min and distilled water for 5 min, followed by 5 baths with PBS for 5 min each. Histological sections were incubated with primary antibodies anti-KIT 1 (Dako Denmark A/S, 1:100) and diluted in PBS buffer containing 1% bovine albumin (BSA; Sigma^®^ A9647) and 0.1% sodium azide (NaN3) for 30 min at 37 °C in an oven, followed by 18 h at 4 °C in a humid chamber (overnight), followed by washing with PBS and incubation with Super Picture Poly HRP conjugate polymer for 30 min in an oven at 37 °C. Revelation was performed using a solution containing diaminobenzene (DAB+Chromogen, Dako Carpinteria, CA, USA). Counterstaining was performed with Hematoxylin. Then, dehydration was performed in alcohol baths (70%, 95% and absolute alcohol twice, with 5 min each), followed by diaphanization with an alcohol solution mixed with xylene, two xylene baths lasting 10 min each, and mounting in synthetic resin and coverslip.

KIT staining patterns and protein localization were assessed as described by Kiupel et al. [22]. KIT protein localization was evaluated by a single veterinary pathologist (M.L.Z.D.). KIT-positive cells were manually counted using light microscopy in a Nikon microscope (40× magnification) and Image Pro Plus software (Image Pro Plus 4.5^®^, Media Cybernetics, Silver Spring, MD, USA). Each MCT was assigned one of the three staining patterns based on the highest staining pattern (staining patterns I, II, or III) present in at least 10% of the neoplastic cell population (estimated based on 100 neoplastic cells in a high-power field) or present in large clusters of neoplastic cells within the tumor [22].

### 2.4. Treatment Protocols

Dogs were randomly assigned to one of two groups, receiving different treatment protocols. Dogs in the IM group (13 animals) received daily oral doses (10 mg/kg) of IM (Gleevec 100 or 400 mg, Novartis Pharma AG, Basel, Switzerland) for 8 weeks. Dogs in the VP Group (11 animals) received 4 weekly, and then 4 biweekly, courses of vinblastine (Faublastina 10 mg, Libbs) given at 2.0 mg/m² by IV bolus injection combined with daily oral prednisone (Meticorten 5 or 20 mg, Schering-Plough S.A., Kenilworth, NJ, USA) administered first at 2 mg/kg, then tapered and discontinued over the course of 12 weeks [20]. The dogs were also prescribed diphenhydramine (2 mg/kg PO, B.I.D.) and omeprazole (0.5 mg/kg PO, once daily) to avoid the effects of possible MCT degranulation.

### 2.5. Treatment Response Assessment

Treatment response was assessed according to the response evaluation criteria in solid tumors established by the Veterinary Cooperative Oncology Group, VCOG [23] and classified as complete response (CR), partial response (PR), stable disease (SD), or progressive disease (PD). Tumor baseline measurements were obtained on day 0 using digital calipers and Computerized Tomography (CT) images (XPRESS/G6 CT Scanner, Toshiba, Tokyo, Japan). The changes in the measurements of the dog’s disease during treatment were checked weekly in the VP group, or every 10 days in the IM group, based on tumor size measurements using a digital caliper. Dogs were excluded from the study if their disease progressed based on digital caliper measurements and received alternative treatment (data not shown).

The objective response rate (ORR) was the primary endpoint of efficacy. ORR was defined as the percentage of evaluable dogs experiencing CR or PR as their best response. The ORR in tumor size measurements on CT images at 8 weeks in the IM group and at 12 weeks in the VP group were compared to those at baseline measurements obtained on day 0 as the primary efficacy endpoint. Tumors were outlined using a semi-automated segmentation algorithm adjusted according to the radiologist’s input, and the longest diameter was measured from the MCT contours. When multiple tumors were present, only the tumor with the largest diameter was considered for the response assessment. ORR was defined as the percentage of evaluable dogs that experienced CR or PR as the best response.

### 2.6. Assessment of the Adverse Events

The assessment of adverse events was based on the criteria established by the Veterinary Cooperative Oncology Group (VCOG-CTCAE) [24]. The dogs in the VP group were checked weekly before drug administration. Dogs in the IM group were rechecked at 10-day intervals. Hematological toxicity, kidney, and liver function were evaluated according to CBC findings, serum creatinine and blood urea nitrogen, and liver enzyme (ALP and AST) levels, respectively. Gastrointestinal toxicity was rated according to the effects reported by the owners. The neutrophil count cutoff for vinblastine administration was set at ≥2500/µL (data not shown).

### 2.7. Statistical Analysis

The variables to be subjected to statistical analysis were classified as numerical or categorical. The numerical data were expressed as means or medians, and categorical data were expressed as frequencies and percentages. The numerical variables were compared using the Mann–Whitney test. Categorical variables were compared between cohorts using Fisher’s exact test. Fisher’s exact test was used to compare the data of overall response (the sum of PR and CR) in IM- or VP-treated, MCT-bearing dogs, and this test was also used to compare the incidence of grade 1 adverse events between IM- and VP-treated dogs. All reported *p*-values are 2-sided, and *p* < 0.05, was used to define statistical significance. All statistical analyses were performed using GraphPad Prism software.

## 3. Results

### 3.1. Patients and Tumor Characteristics

Twenty-four dogs from 10 different breeds (Boxer, Siberian Husky, Labrador, Bulldog, Bernese, Yorkshire, Pit Bull, Golden Retriever, Rottweiler, Dogo Argentino) and mongrels, aged 2 to 16 years (mean age, 8 years; 12 males and 12 females), were included in the study. The characteristics of the dogs in the IM and VP groups are shown in Table 2. All MCT cases included in the study were cutaneous. There was no statistical difference between the breeds, genders, ages, localization, WHO staging and metastasis of the dogs involved in the study when the two treatment groups were considered. (Table 3).

Four out of thirteen dogs from the IM group and two out of eleven dogs from the VP group presented metastasis to lymph nodes at the time of diagnosis (Table 3). Only the target lesions were considered for the evaluation of the IM or VP treatments.

The histological characteristics of the MCT samples from the dogs included in this study are shown in Table 4. In the IM group, 100% of the cases were grade 2 and low grade according to Patnaik’s and Kiupel’s grading systems, respectively. In the VP group, 90.90% of the MCTs were grade 2 and 1 MCT was grade 3; 1 MCT was grade 3 and high grade. Tumor grading did not differ significantly between the groups (Patnaik and Kiupel classifications: *p* > 0.999 and *p* = 0.409).

### 3.2. KIT Protein Localization

**Figure 1:**
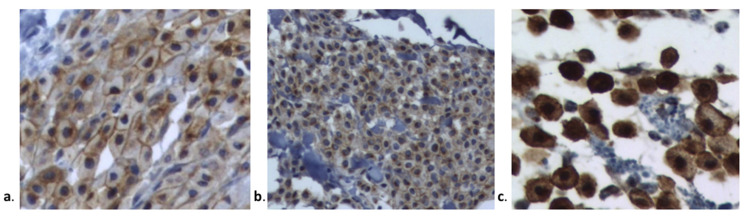
Kit staining patterns according to Kiupel et al., 2004 [19] (**a**)**.** Kit I: membrane-associated staining, (**b**)**.** Kit II: focal to stippled cytoplasmic staining with decreased membrane-associated staining, and (**c**)**.** Kit III: diffuse cytoplasmic staining.

KIT protein localization was determined in all MCTs by microscopic examination. The KIT staining patterns are presented in Figure 1 and Table 4. Most MCTs in groups IM and VP were classified according to the Kit 2 pattern (61.50% and 63.63%, respectively). The KIT pattern did not differ significantly between groups (*p* = 0.999).

### 3.3. Treatment Response

Response to treatment was assessed from the CT scan images of tumors using the unidimensional rules (longest diameter), according to the VCOG [23] criteria.

Among the 13 MCT-bearing dogs treated with IM, one achieved CR, three achieved PR, five achieved SD, and four had PD (7.7%, 23.1%, 38.5%, and 30.8%, respectively), yielding an overall response rate (the sum of CR and PR) of 30.76% (Table 5). Regarding the 11 VP-treated dogs with MCT, none achieved complete remission (CR): one dog had a partial response (PR) (9.09%), six dogs had stable disease (SD), and four animals had progressive disease (PD). The treatment response data are presented in Table 2 and Table 5.

When the overall response to IM or VP treatments was considered, that is, the sum of the complete response (CR) + partial response (PR), were compared in a contingency table and analyzed with the Fisher exact test, the difference was highly statistically significant (*p* = 0.0003). Thus, IM determined a higher overall response in these cases compared with VP.

### 3.4. Adverse Events

Adverse events in patients treated with imatinib mesylate (IM), vinblastine, and prednisone (VP) were classified according to the VCOG-CTCAE [24] and are depicted in Table 6. Adverse events in patients treated with IM were all classified as grade 1, while those in VP animals were classified as grades 1 and 2; 38.46% of IM group dogs and 54.54% of the VP group had grade 1 adverse events, and one dog in the VP group had grade 2 adverse events.

When grade 1 event incidence in IM dogs was compared with that in VP dogs, the difference was statistically significant: VP dogs had a statistically higher incidence of grade 1 events.

## 4. Discussion

Since mast cell tumors are very prevalent tumors in dogs, well-tolerated and efficacious treatments are welcomed. Therefore, the investigation of targeted therapies may be beneficial to dogs, as these therapies are efficacious in humans. VP is the standard adjuvant chemotherapy regimen after the surgical resection of canine MCTs and the regimen of choice for the treatment of non-resectable tumors or advanced disease [26,27]. In this study, vinblastine was administered at doses commonly reported for canine MCT treatment (2 mg/m^2^) [28,29]. To the best of our knowledge, there are no other reports in the literature regarding comparisons between IM and VP treatments for canine MCT, highlighting the importance of this study. Previous studies indicated that IM might have a biological activity in some dogs with MCT [13,30,31,32]; therefore, we decided to test whether IM was more beneficial to dogs bearing MCT than those bearing VP.

The dogs included in this study were obtained from the Veterinary Teaching Hospital (HOVET) of the School of Veterinary Medicine and Animal Science of the USP. The 24 dogs bearing mast cell tumors were assigned to two groups of dogs according to predefined inclusion criteria; thus, 13 animals were treated with the IM protocol and 11 animals received conventional treatment with VP. The two groups of dogs showed comparable breed, gender, age, localization of the tumors, and WHO staging, as revealed by the absence of statistical significances in the comparisons. All MCT samples were subjected to a histopathological analysis to confirm the diagnosis and to assign a grading score according to the two available systems (Patnaik and Kiupel). In addition, all MCTs were subjected to immunohistochemical analysis for c-KIT to evaluate their KIT patterns. Again, the MCT cases included in this study had similar grades and KIT staining patterns. Through the statistical comparisons, it was possible to verify that the MCT-bearing dogs assigned to the two treatment groups started from very similar conditions. This is very important when comparisons between two treatments are made.

The dogs that received the IM or VP protocols had their tumors initially measured using digital calipers and/or CT scanning, and they were subsequently measured at defined time points. Both groups of dogs contained animals with PR, SD, and PD according to the VCOG evaluation protocol [23]; however, only in the IM group, one animal presented complete remission. The objective response rate (ORR) was then calculated by summing the percentage of dogs that had a complete response (CR) plus the percentage of dogs with a partial response (PR) in each treatment group. Interestingly, the IM group showed a significantly higher ORR than the VP group.

Imatinib (STI-571/Gleevec^®^) is a 2-phenylamino-pyrimidine compound and is a selective inhibitor of the Abl tyrosine kinase enzyme and the BCR-ABL gene. This drug acts as a specific competitor of the cellular ATP receptor of the tyrosine kinase domain of Abl and prevents the ability of this protein to transfer ATP phosphate groups and phosphorylated tyrosine residues, which prevents the transduction of energy signals necessary for cell proliferation and apoptosis. IM was approved by the FDA in 2002 for the treatment of inoperable and/or metastatic malignant gastrointestinal stromal tumors (GIST) and chronic myeloid leukemia (CML) in humans [33].

In 2003, London et al. described internal tandem duplications in exons 11 and 12 of c-kit in the mast cell tumors of dogs [34]. The first use of imatinib mesylate in veterinary medicine was reported by Isotani et al, 2006 [35], when they identified a c-kit internal tandem duplication in exon 8 in a feline mast cell tumor.

Kobie et al., 2007 [36], first showed that IM was effective against canine mast cell tumors in mouse xenograft models, and stated that canine MCTs could be a potential target for imatinib therapy. In addition, IM showed clinical activity against MCT in 21 dogs, but the response could not be predicted based on the presence or absence of a mutation in exon 11 of c-kit [13]. IM also elicited a clinical response in a canine case of MCT via inhibition of the constitutively activated KIT, caused by a c-kit c.1523A > T mutation [31]. IM also induced caspase-dependent apoptosis in canine neoplastic mast cells possessing mutations in c-kit exon 11 [37] and demonstrated an effective response in two dogs bearing c-kit mutations in exon 11 [32]. IM was successfully used to treat a dog with gastrointestinal c- stromal tumors with a kit mutation [38].

In our study, the majority of the mast cell tumors had their DNA extracted and amplified by PCR for exons 10 and 11; mutations were found in four patients (two from the IM and two from the VP group), characterized by base substitutions near the *3*′ splice site of exon 11, position 1759 in codon 576 (data not shown). Activating internal tandem duplication mutations were not found in this study. Mutations were not associated with the response to treatment. This is in accordance with previous studies of IM [13] or toceranib [39].

Adverse events in the two groups of MCT-bearing dogs were scored and recorded. Most dogs in both groups had grade 1 adverse events according to the VCOG-CTCAE [24], and only one animal from the VP group had a grade 2 adverse event. When the percentage of dogs that showed grade 1 events was compared in both groups, the percentage of dogs in the VP group was significantly higher. The adverse effects of VP at the doses used in this study were deemed mild and acceptable, reflecting primarily hematological and gastrointestinal toxicity, as reported elsewhere [27,28,29]. Dogs treated with IM in this study had varying degrees of gastrointestinal toxicity and mild hematological toxicity. These adverse effects were lower than those caused by the VP chemotherapy. Generally, IM is mild (grade 1) to moderate (grade 2) in intensity, transient, and medically manageable [31], which suggests that it is a safer alternative to canine MCT. VP treatment is in generally well tolerated, but the dose intensity must be well adjusted for the treatment of canine MCT in order to avoid unwarranted toxicity [26]. As expected, the targeted therapy with IM determined less severe adverse events.

The relatively low number of dogs included in the study may be considered a limitation. However, the homogeneous groups of dogs treated with IM or VP, in which no statistical differences in breeds, genders, ages, WHO staging or grading and KIT patterns were found, may minimize this limitation and create confidence in the statistical differences between treatments and adverse events.

Another limitation refers to the treatment duration in IM and VP groups. Although dogs were treated with IM for 8 weeks, the VP protocol has a duration of 12 weeks. Even with these differences in treatment length, the IM treatment was more successful within 8 weeks regarding the outcome than the VP that took 12 weeks, and IM animals had a better ORR. This may represent an additional advantage of the use of IM.

We did not include the KIT sequencing of the MCTs in this report, although it was performed in the majority of studies. As stated by Willmann et al., 2021 [40], despite the fact that a number of KIT mutations were detected in canine MCTs, the KIT sequencing approach has not yet been adopted in the routine of veterinary oncology, and controversial data were recently presented, showing that KIT mutations do not correlate with the response to tyrosine kinase inhibitors or even might have a worse outcome compared to dogs with wild-type KIT MCT. Since the sequencing results of exons 10 and 11 only are available in our study (data not shown), this is a limitation that will be undoubtedly avoided by our group in future studies involving canine MCTs.

## 5. Conclusions

In conclusion, the aim of this study was to compare standard VP polychemotherapy and targeted therapy with IM in cases of mast cell tumors, and to investigate the associations between treatment response and factors that are known to impact MCT progression, such as histologic grade and KIT pattern. Although other tyrosine-kinase inhibitors are currently available to treat MCT, including toceranib (Palladia^®^) and masitinib (Masivet^®^), IM is a current therapy used in humans bearing tumors, which can also be used to treat dogs bearing MCT. The goal of any clinical study in veterinary oncology is to find better treatments for current diseases, which focus on efficacy and safety. Our study has shown that IM presents some advantages compared to conventional chemotherapy and may be used for the benefit and comfort of dogs with low-grade MCT.

## Figures and Tables

**Table 1:** Inclusion and exclusion criteria for dogs bearing MCTs.

Inclusion Criteria	Exclusion Criteria
Diagnosis of inoperable MCTs	Dogs with findings suggestive of cardiac, kidney, or liver diseases
MCTs localized in sites not amenable to surgical resection	Concomitant neoplasms
Eligibility to receive chemotherapy with IM or VP	Concurrent diseases requiring immunosuppressive therapy (i.e., severe atopic or immune-mediated disease) other than prednisone
	Concurrent systemic antineoplastic therapy
	MCTs with systemic spread
	Involvement of more than one lymph node
	Dogs weighing less than 5 kg or intended for breeding

**Table 2:** Characteristics of the dogs included in the study and their responses to imatinib mesylate (IM) or vinblastine and prednisone (VP).

Dog	Sex	Age (Years)	Breed	Metastasis	Target Tumor	WHO Stage *	Grade (Patnaik/ Kiupel)	KIT Staining Pattern **	% Variation of the Longest Diameter (Target Lesion)	Response According to VCOG ***
	Imatinib Mesylate (IM)									
C5IM	M	7	Boxer	Absent	Genital /perianal	IIIa	2/Low	2	13	SD
C6IM	M	8	Mongrel	Absent	LHL	Ia	2/Low	2	21.1	PD
C11IM	F	12	Siberian Husky	Absent	Head/Neck	IIIb	2/Low	2	23.3	PD
C12IM	F	8	Labrador	Absent	RHL	Ia	2/Low	2	−43.2	PR
C14IM	M	2	Bulldog	Absent	RHL	Ia	2/Low	2	7.5	SD
C15IM	F	10	Boxer	Mandibula lymph node	Head/Neck	IIIb	2/Low	3	−100	CR
C17IM	F	5	Bernese	Cervical lymph node	RHL	IIa	2/Low	3	−40.8	PR
C22IM	M	9	Yorkshire Terrier	Inguinal lymph node	Genital/perianal	IIIa	2/Low	1	77.7	PD
C25IM	F	6	Mongrel	Absent	Trunk	IIIa	2/Low	2	−15.3	SD
C27IM	F	16	Mongrel	Cervical lymph node	LFL	IIIb	2/Low	3	33.07	PD
C28IM	F	13	Mongrel	Absent	Trunk	IIIb	2/Low	3	−16.8	SD
C29IM	M	8	Yorkshire Terrier	Absent	LHL	Ia	2/Low	2	−55.9	PR
C30IM	M	8	Mongrel	Absent	LHL	Ia	2/Low	2	−4.39	SD
	Vinblastine and Prednisone (VP)									
C4VP	M	7	Mongrel	Absent	Trunk	Ia	2/Low	3	0.2	SD
C7VP	F	10	Mongrel	Absent	RHL	IIIa	2/Low	2	30.9	PD
C8VP	F	10	Rotweiler	Absent	RHL	IIIa	2/Low	2	20.7	PD
C9VP	M	10	Golden Retriever	Absent	Genital/perianal	IIIa	2/Low	2	−22.7	SD
C10VP	M	4	Boxer	Absent	Trunk	Ia	2/Low	3	−14.9	SD
C16VP	F	9	Pit bull	Absent	Trunk	IIIa	2/Low	2-3	−15.1	SD
C19VP	F	5	Golden Retriever	Absent	Head/Neck	Ia	2/Low	2	14.7	SD
C21VP	F	6	Boxer	Absent	LHL	Ia	2/Low	1	−13.8	SD
C23VP	M	8	Labrador	Inguinal lymph node	Genital/perianal	IIb	3/High	2	43.9	PD
C24VP	M	9	Labrador	Satellite lesions	Trunk	IIIa	2/Low	2	27.2	PD
C26VP	M	7	Dogo Argentino	Popliteal lymph node	LHL	IIIa	2/Low	3	−42.1	PR

LHL = left hind limb; RHL = right hind limb; LFL = left fore limb. * WHO Staging system—Owen, 1980 [25]. ** Classification of the staining pattern was done according to: (1) Membrane-associated staining, (2) Focal to stippled cytoplasmic staining with decreased membrane-associated staining; and (3) diffuse cytoplasmic staining. *** CR = complete response, PR = partial response, SD = stable disease, and PD = progressive disease.

**Table 3:** Demographics of animals enrolled into the study comparing IM and VP treatments in dogs bearing mast cell tumors.

Treatment Groups	IM	VP		*p*-Value
Breeds	Number (%)	Number (%)	Total Number (%)	*p* = 0.4321
Mongrel	5 (38.46)	2 (18.18 %)	7 (29.17)	
Boxer	2 (15.38)	2 (18.18)	4 (16.67)	
Labrador	1 (7.69)	2 (18.18)	3 (12.50)	
Yorkshire	2 (15.38)	0 (0)	2 (8.33)	
Golden Retriever	0 (0)	2 (18.18)	2 (8.33)	
Siberian Husky	1 (7.69)	0 (0)	1 (4.17)	
Bulldog	1 (7.69)	0 (0)	1 (4.17)	
Bernese	1 (7.69)	0 (0)	1 (4.17)	
Pitbull	0 (0)	1 (9.09)	1 (4.17)	
Rottweiler	0 (0)	1 (9.09)	1 (4.17)	
Dogo Argentino	0 (0)	1 (9.09)	1 (4.17)	
Total = 11 breeds	13 dogs	11 dogs	24	
Gender	Number (%)	Number (%)	Total Number (%)	
M	6 (46.15)	6 (54.54%)	12 (100.69)	
F	7 (53.84)	5 (45.45%)	12 (99.29)	*p* = 0.3222
Total	13 (100)	11 (100%)	24 (200)	
Age	8.615 + 3.595	7.727 + 2.102		*p* = 0.479
Localization	Number (%)	Number (%)	Total Number (%)	*p* = 0.889
Genital/perianal	2 (15.38)	2 (18.18)	4 (16.67)	
Head/neck	2 (15.38)	1 (9.09)	3 (12.50)	
LHL	3 (23.07)	2 (18.18)	5 (20.83)	
RHL	3 (23.07)	2 (18.18)	5 (20.83)	
LFL	1 (7.69)	0 (0)	1 (4.16)	
Trunk	2 (15.38)	4 (36.36)	6 (25)	
Total	13	11	24	
Metastasis	Regional lymph nodes in 4/13 cases (30.77%)	Regional lymph nodes in 3/11cases (27.27%)		
	Absent in 9/13 dogs	Absent in 8/11 dogs		
WHO stage	Ia 5/13 (38.46%)	Ia 4/11 (36.36%)		*p* = 0.6286
	IIa 1/13 (7.69%)	IIIa 6/11 (54.54%)		
	IIIa 3/13 (23.07%) Ib 4/13 (30.77%)	IIb 1/11 (9.09%)		
TOTAL	13	11	24	

LHL= left hind limb; RHL = right hind limb; LFL = left fore limb.

**Table 4:** Histological characteristics of the MCT in dogs assigned to the two treatment groups.

	IM	VP	Statistics
Grading Systems			
Patnaik grades	Grade 1–0 Grade 2 (13/13—100%) Grade 3–0	Grade 1–0 Grade 2 10/11 (90.90%) Grade 3 1/11 (9.09%)	*p* > 0.999 (Patnaik’s) and *p* = 0.409 (Kiupel)
Kiupel tiers	Low (13/13–100%) High–0	Low (10/11–90.90%) High (1/11–9.09%)	
KIT pattern			
KIT I	1/13 (7.69%)	1/11 (9.09%)	*p* = 0.999
KIT II	8/13 (61.5%)	7/11 (63.63%)	
KIT III	4/13 (30.79%)	3/11 (27.27%)	
Total	13 dogs	11 dogs	

**Table 5:** Target lesion response.

Treatment Group/Target Lesion Response *	IM Number of Dogs/Total (%)	VP Number of Dogs/ Total (%)
Partial response (PR)	3/13 (23.07%)	1/11 (9.09%)
Complete response (CR)	1/13 (7.69%)	none
Stable disease (SD)	5/13 (38.46%)	6/11 (54.54%)
Progressive disease (PD)	4/13 (30.80%)	4/11 (36.36%)
Objective response rate (ORR)(PR + CR)	4/13 (30.76%) *	1/11 (9.09%)

* According to VCOG [21] Stable disease (SD): less than 30% reduction (PR) or 20% increase (PD) in the sum of diameters of target lesions, taking as a reference the smallest sum of diameters in the study; partial response (PR): at least a 30% reduction in the sum of diameters of target lesions, taking as a reference the baseline sum; progressive disease (PD): either the appearance of one or more new lesions or at least a 20% increase in the sum of diameters of target lesions, taking as a reference the smallest sum on study. The total also showed an absolute increase of 5 mm. Complete response (CR): disappearance of all target lesions. Pathologic LNs. * *p* = 0.0003 when compared to VP.

**Table 6:** Adverse events in patients treated with imatinib mesylate (IM) or vinblastine and prednisone (VP) according to VCOG-CTCAE [24].

Adverse Event *	Grade 1		Grade 2		Grades 3–5	
	IM	VP	IM	VP	IM	VP
Leucopenia	1/13 (7.69%)	2/11 (18.18%)	-	-	-	-
Diarrhea	1/13 (7.69%)	-	-	-	-	-
Vomiting	1/13 (7.69%)	1/11 (9.09%)	-	1/11 (9.09%)	-	-
Weight loss	-	-	-	-	-	-
Dysorexia/anorexia	1/13 (7.69%)	3/11 (27.27%)	-	-	-	-
Renal toxicity	-	-	-	-	-	-
Lethargy	-	-	-	-	-	-
Dermatologic	1/13 (7.69%)	-	-	-	-	-
TOTAL	5/13 (38.46%)	6/11 (54.54%) **	0	1/11 (909%)	0	0

* [24]. ** significantly higher than in IM dogs (*p* = 0.0331) (Fischer exact test).

## Data Availability

Data are available upon request.
